# The complexity of selection at the major primate β-defensin locus

**DOI:** 10.1186/1471-2148-5-32

**Published:** 2005-05-18

**Authors:** Colin AM Semple, Alison Maxwell, Philippe Gautier, Fiona M Kilanowski, Hayden Eastwood, Perdita E Barran, Julia R Dorin

**Affiliations:** 1 MRC Human Genetics Unit, Western General Hospital, Edinburgh, EH4 2XU, UK; 2 School of Chemistry, The University of Edinburgh, The King's Buildings, West Mains Road, Edinburgh, EH9 3JJ, UK

## Abstract

**Background:**

We have examined the evolution of the genes at the major human β-defensin locus and the orthologous loci in a range of other primates and mouse. For the first time these data allow us to examine selective episodes in the more recent evolutionary history of this locus as well as the ancient past. We have used a combination of maximum likelihood based tests and a maximum parsimony based sliding window approach to give a detailed view of the varying modes of selection operating at this locus.

**Results:**

We provide evidence for strong positive selection soon after the duplication of these genes within an ancestral mammalian genome. Consequently variable selective pressures have acted on β-defensin genes in different evolutionary lineages, with episodes both of negative, and more rarely positive selection, during the divergence of primates. Positive selection appears to have been more common in the rodent lineage, accompanying the birth of novel, rodent-specific β-defensin genes. These observations allow a fuller understanding of the evolution of mammalian innate immunity.

In both the rodent and primate lineages, sites in the second exon have been subject to positive selection and by implication are important in functional diversity. A small number of sites in the mature human peptides were found to have undergone repeated episodes of selection in different primate lineages. Particular sites were consistently implicated by multiple methods at positions throughout the mature peptides. These sites are clustered at positions predicted to be important for the specificity of the antimicrobial or chemoattractant properties of β-defensins. Surprisingly, sites within the prepropeptide region were also implicated as being subject to significant positive selection, suggesting previously unappreciated functional significance for this region.

**Conclusions:**

Identification of these putatively functional sites has important implications for our understanding of β-defensin function and for novel antibiotic design.

## Background

Antimicrobial peptides have a critical role in the vertebrate innate immune defence against microbes. These peptides have potential as therapeutics and intelligent drug design relies on understanding how these molecules function. Defensins are peptides, which are generally cationic, are produced as prepropeptides and can be divided into subclasses based on the distribution of the six canonical cysteines that are located in the mature peptide. There are only two subclasses shared between mouse and human, the α and β-defensins. These molecules both have six canonical cysteine residues but differ in the spacing of these residues and the intramolecular disulphide bridges formed [1]. The antimicrobial activity of both α and β-defensins *in vivo *is well established [2]. More recently β-defensins have been shown to act as a link between adaptive and innate immunity [3] and play important roles in cancer progression [4]. This has stimulated great interest in the function and evolution of β-defensins in primate lineages [5].

Genes that are involved in host defence often display high rates of genomic divergence and evidence for adaptive evolution. As seems to be the case with other proteins involved in the immune response, such as MHC molecules, immunoglobulins and α-defensins, this selection may be a response to the rapid evolution of pathogens [6-8]. In agreement with this, the four well-studied human β-defensins vary in their expression patterns as well as their antimicrobial and antiviral activities [5]. We have previously investigated the 8 functional human genes at the major 8p22-p23 β-defensin locus and 11 genes at the orthologous mouse locus. In both mouse and human, β-defensin paralogues show little sequence similarity in the mature peptide region and this divergence appears to have been driven by positive selection following duplication [9,10]. These genes show an unusual pattern of evolution, with rapid divergence between second exon sequences that encode the mature peptides matched by relative stasis in the first exons that encode signal peptides [10]. However, these previous studies detected positive selection acting during the more distant evolutionary history of this locus to produce a diverse cluster of paralogous genes apparently established early in mammalian evolution [9,10]. The evidence for positive selection acting within primate lineages since this paralogous cluster was established has been more equivocal and fragmentary. It has been reported that both DEFB1 [11] and DEFB103 (formerly DEFB3) [12] have evolved neutrally in primate lineages with no evidence for positive selection. In contrast there is circumstantial evidence to suggest that the evolution of primate DEFB4 (formerly DEFB2) genes has involved positive selection [13]. The selective forces operating on the other β-defensins at this locus in primate lineages have, until now, remained unknown.

Many of the previous analyses of β-defensin genes depended upon statistical tests based on a traditional pairwise approach, calculating and comparing the rate of non-synonymous (dN) and synonymous (dS) substitution between two sequences averaged over all codons. However this approach is not appropriate for short sequences (such as β-defensins) since they do not provide sample sizes (numbers of sites and/or substitutions) that are large enough to give significant results. In addition, because such methods are based upon the dN/dS ratio across the whole length of the sequences under consideration, purifying selection at some sites could obscure the action of positive selection at other sites. A popular alternative strategy is to use likelihood ratio tests (LRTs) that allow one to estimate the dN/dS ratio (ω) at particular sites rather than averaging it over the whole molecule. This site-specific analysis has been successful at detecting positive selection in a variety of genes, particularly in gene families following expansion by duplication, and computer simulations have confirmed the power of the analysis [14-16]. However, Suzuki and Nei [17-19] found that positively selected amino acid sites are more reliably inferred by parsimony-based methods than by likelihood-based methods, with the latter prone to producing false positives. Recently a new sliding window approach based on maximum parsimony has been devised to conservatively predict the presence of selection from alignments, with special attention paid to reducing false positives [20]. Also Suzuki [21] has developed software that can detect positively and negatively selected sites based upon maximum parsimony, Bayesian or maximum likelihood methods. Given the known shortcomings of the methods available it seems most prudent to restrict attention to sites that are inferred to be subject to selection by multiple methods [22,23]. We have used a combined strategy, complementing likelihood and parsimony-based approaches to give a comprehensive account of the selection acting at the main β-defensin locus in human, and the orthologous loci in a range of other primates and mouse. We provide statistically significant evidence for the action of both positive and negative selection in both rodent and primate lineages, and reveal the putatively functional sites within the peptide structures that have been subject to these forces at different times.

## Results

**Figure 1:**
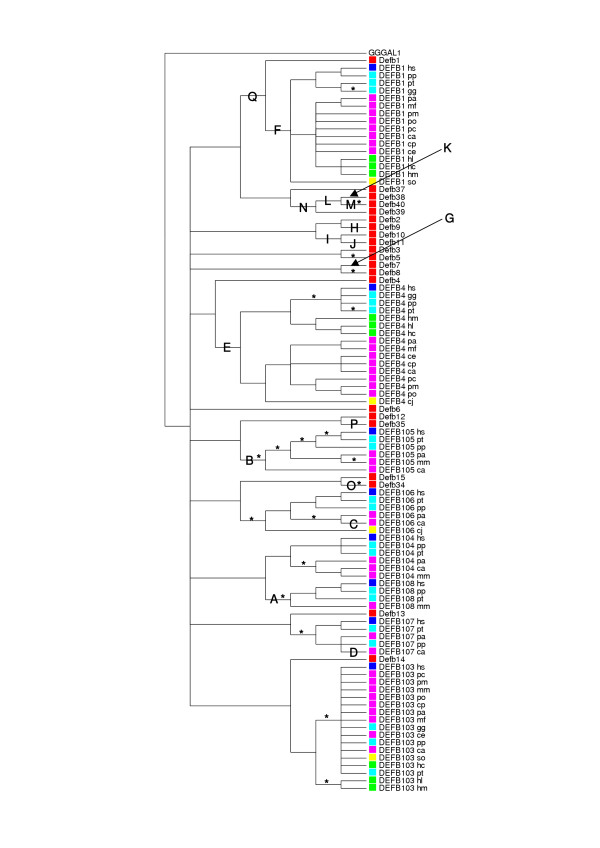
**Phylogenetic tree relating primate and mouse β-defensin proteins constructed using neighbour-joining. **Branches with less than 50% bootstrap support have been collapsed. Species and families of interest are coloured as follows: mouse sequences are red, human sequences are dark blue, Hominidae other than human are light blue, Cercopithecidae are purple the Hylobatidae are green and the Callitrichidae are yellow. Primate species names are abbreviated as detailed in Materials and Methods, mouse genes are in lower case. Branches labelled with letters show significant (P < 0.0001) evidence of positive selection (see Figure 3 and Additional Files 3 and 4 details) and asterisks indicate branches showing significant (P < 0.0001) evidence for negative selection.

A neighbor-joining (NJ) tree was constructed from the 97 aligned mouse and primate amino acid sequences using p-distance estimates (Figure 1; full branch length and bootstrapping support annotation for this tree are available in Additional Files). This tree and an alignment of nucleotide sequences derived from the protein alignment (see supplementary material in Additional Files) were used in the analyses that follow. It should be noted that all 21 mouse genes analysed here are readily detectable on the orthologous rat chromosome 16 and that apparently mouse specific clades in Figure 1 are therefore likely to be rodent specific. Orthologs of the genes within all rodent specific clades in the tree (Defb37/38/39/40, Defb2/9/10/11, Defb7/8, Defb3/5 and Defb6) were not detectable in searches of the whole genome shotgun sequence data for the dog genome. (Rat and dog genes were not analysed in detail due to the gapped and potentially misassembled nature of these draft genomes.) In contrast orthologs of all 8 primate genes were readily detectable in the dog genome sequence data (implying that they are more than 90 million years old), which supports the conclusion that the apparently rodent specific clades have indeed arisen more recently in the rodent lineage.

### Positive selection has acted in both primate and rodent lineages

**Figure 4:**
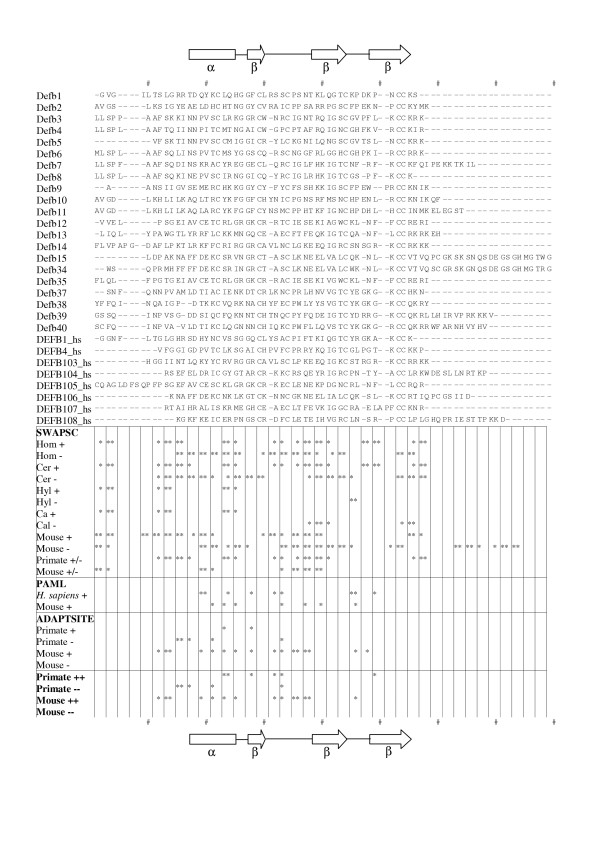
**Alignment of human and mouse β-defensin peptides showing sites of selection. **Sites of significant positive and negative selection (according to SWAPSC, PAML and ADAPTSITE) in primate and mouse lineages are indicated by asterisks. Hashes indicate every tenth position in the alignment. Hominidae, Cercopithecidae, Hylobatidae and Callitrichidae are abbreviated to Hom, Cer, Hyl and Cal respectively. The positions of the beta strands and alpha helix in DEFB1 are indicated, the residues within the prepropeptide region are those before the alpha helix. The horizontal lines under the alignment denote the results of different analyses: SWAPSC results for primate and mouse lineages individually, SWAPSC sites under positive and negative selection in different branches within primates (Primate +/-) and mouse (Mouse +/-), PAML results for sites under positive selection, ADAPTSITE results for sites under positive an negative selection, sites implicated as under positive selection by two or more different programs for primate (Primate ++ and Primate --) and mouse (Mouse ++ and Mouse--) lineages.

Significant evidence of selection was sought using three different programs: PAML, ADAPTSITE and SWAPSC. Three pairs of PAML site-specific likelihood models were compared that assume variable selective pressure (as determined by the value of ω) among sites: M0 (one-ratio) and M3 (discrete), M1 (neutral) and M2 (selection), and M7 (beta) and M8 (beta&.). The M3 model (allowing variation in ω between two site classes) was a significantly better fit to the data than M0 (allowing no variation in ω) with the LRT statistic as follows: 2Δl = 2(-3468.13 -(-3260.32) = 415.62, P < 0.0001 with 2 degrees of freedom (df). However subsequent Bayesian analysis failed to identify any sites under positive selection with greater than 95% confidence. The LRT between M1 and M2 failed to show a significant difference in fit to the data. Model M7 assumes a beta distribution for ω over 10 categories of sites. The beta distribution is limited to values between 0 and 1 providing the most flexible null hypothesis, and most stringent test, for testing positive selection. Model M8 adds another site class to the M7 model, within which ω is estimated from the data. The M8 model suggested that a small proportion of sites were under strong positive selection (ω = 21.82), but again no specific sites were implicated as under positive selection in the subsequent Bayesian analysis (95% threshold). An LRT showed that the M8 model allowing positive selection was a significantly better fit to the data than M7: 2Δl = 2(-3260.66 -(-3253.50) = 16.32, P < 0.001 with 2 degrees of freedom (df). In addition, M8 was the best fit to the data of all 6 site-specific models tested. These somewhat equivocal results are not unexpected for this data set. It is known that these LRTs suffer from a lack of power to detect significant effects when divergence between sequences in the data set is low [15]. The levels of divergence between many of the primate sequences in this data set are often very low, and occasionally zero. Nevertheless the LRTs suggested that the best description of these data is a model incorporating many categories of variable ω including one showing positive selection. However, using this dataset, PAML could not confidently (i.e. at greater than 95% confidence) suggest the particular sites subject to positive selection. An earlier analysis of the 8 paralogous human β-defensins alone (and therefore based upon a set of sequences with higher average pairwise divergence), and using the same LRTs, demonstrated significant evidence for the operation of positive selection and 9 sites were nominated in more than one model [10]. The locations of these sites are shown in Figure 4.

To complement the analysis of Semple et al. [10] the same site-specific LRTs were applied to an alignment of the mouse genes alone. In each case the models consistent with the presence of sites under positive selection were significantly better fits to the data than the paired null models. M3 was a better fit to the data than M0 with an LRT statistic 2Δl = 2(-2716.01 -(-2563.54)) = 304.94 and P < 0.001 with 2 df. M2 was a better fit to the data than M1: 2Δl = 2(-2603.43 -(-2569.69) = 67.50 and P < 0.001 with 2 df. M8 was a better fit to the data than M7: 2Δl = 2(-2575.43 -(-2559.81) = 31.25, P < 0.001 with 2 df. Again, M8 was the best fit to the data of all 6 site-specific models tested. In summary, these LRTs indicated that ω varies significantly between sites among these mouse genes, and in every LRT the parameters estimated suggested a substantial proportion of sites are under positive selection. The parameters estimated were fairly consistent, with ω estimated to be between 1.84 and 3.68 and with this positive selection acting upon 34–50% of sites. Seven particular sites were consistently implicated (in M2, M3 and M8 models) as being under positive selection (Figure 4) with greater than 95% confidence.

The ADAPTSITE analysis of these data was in general agreement with the LRT results described above: all three approaches (maximum parsimony, distance-based and maximum likelihood) estimated a minority of sites (1–5% in primate sequences and 2–8% in mouse) where dN > dS. However only the likelihood approach yielded statistically significant evidence for positively selected sites (1% in primate sequences and 8% in mouse). This may be explicable by the greater sensitivity of the likelihood approach [21]. All three ADAPTSITE approaches showed significant evidence for negative selection in a minority (3–5% in primate sequences and 0–8% in mouse) of sites. The general picture that emerges from the ADAPTSITE analysis suggests that positive selection has been more important than negative selection in mouse lineages and that the opposite is true of primate lineages.

The SWAPSC sliding window analysis of all mouse and primate data also broadly reflected the LRT results: the data set was estimated to contain sites subject to a wide range of ω values, including a small number under positive selection. Specifically 0.77% of the sites were estimated to be subject to positive selection and 1.15% to negative selection. The branches identified as under positive and negative selection are indicated in Figure 1 and reveal the dynamic evolutionary history of this locus. Of the 8 primate genes examined, positive selection has played a role in the evolution of 6 and negative selection has acted upon all 8. However the 21 genes at the orthologous rodent locus appear to have less turbulent histories, with 10 and 4 genes subject to positive and negative selection respectively. This leaves 7 mouse genes lacking significant evidence of either positive or negative selection. It is also notable that the majority (7/11) of mouse genes that have experienced detectable selection belong to apparently rodent specific clades in Figure 1 (clades containing rodent genes not present in the human genome or in the whole genome shotgun sequence data for the dog genome).

### Selection in β-defensins varies spatially and temporally

**Figure 2:**
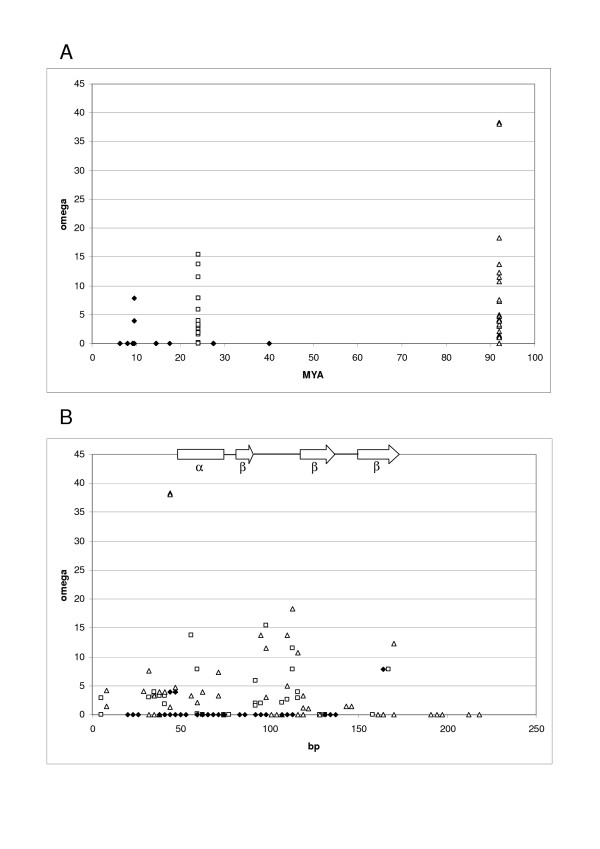
**Selection across evolutionary time and sequence space. **The graphs show data for primate (black diamonds), mouse (white squares) and more ancient branches (white triangles) for all significant positive and negative selection detected A: values of omega and dates. B: values of omega across the alignment in bp (midpoints of 3 codon windows). The positions of the beta strands and alpha helix in DEFB1 are indicated.

The branches in the tree in Figure 1 can be divided into three categories: (i) primate branches (i.e. those relating only primate orthologs that diverged ~5–40 million years ago); (ii) rodent branches (i.e. those among rodent genes that diverged ~12–24 million years ago (MYA) but absent from the human and dog genomes); (iii) more ancient branches (i.e. those leading to clades containing primate and rodent genes or those leading to primate clades that possess rodent or dog orthologs indicating events ~40–92 million years ago). Figure 2A provides an overview of the evolutionary dynamics within these three broad categories according to SWAPSC. It is clear that the selective episodes affecting primate genes have involved relatively low values of ω with many periods of negative selection while those affecting rodent genes have spanned a broader range of ω with few episodes of negative selection. More ancient branches seem to have involved the highest values of ω, which is consistent with the view that the early stages of duplication and diversification among mammalian β-defensin paralogs involved strong selection. The later stages of evolution within mammalian groups, and particularly primates, seem to have involved less innovation. Figure 2B shows that the focus of most positive selection in rodent and ancient branches but also of negative selection in primate branches has been the first ~120 bp of the alignment. These first 40 amino acids include the alpha helix and first beta strand of the mature defensin peptide [24].

SWAPSC analysis suggests that in both primate and rodent lineages there have been episodes of selection in historically consecutive branches that have affected the same regions of these genes. For example in the primate lineage there was an episode of positive selection ~40–92 MYA (branch E in Figure 1) which was followed by an episode of negative selection within the past ~14.5 million years (MY), with both episodes affecting positions 109–117 in the alignment. Similarly, a burst of positive selection during the evolution of the common ancestor of primate and rodent DEFB1 (branch Q in Figure 1) was followed by negative selection at the same site (positions 94–102 of the alignment) within the past 8.09 MY of primate evolution. Primate evolution has also been marked by bouts of recurring negative selection at the same site, most clearly in the case of DEFB105. Here a site (positions 55–63 in the alignment) within the alpha helix has been subject to negative selection on four occasions during the divergence of old world monkeys (OWM) from *H. sapiens *and *P. troglodytes*. This contrasts with the rodent lineage where only consecutive bursts of positive selection are seen to affect the same sites. Within the rodent specific clade containing Defb38/39/40 two different sites are affected by positive selection, and each site has been targeted by selection at two points during their evolutionary past: positions 31–39 in the prepropeptide region at branches N and K; positions 109–120 in the second beta strand at branches L and M.

### Sites of ancient and relatively recent selection

**Figure 3:**
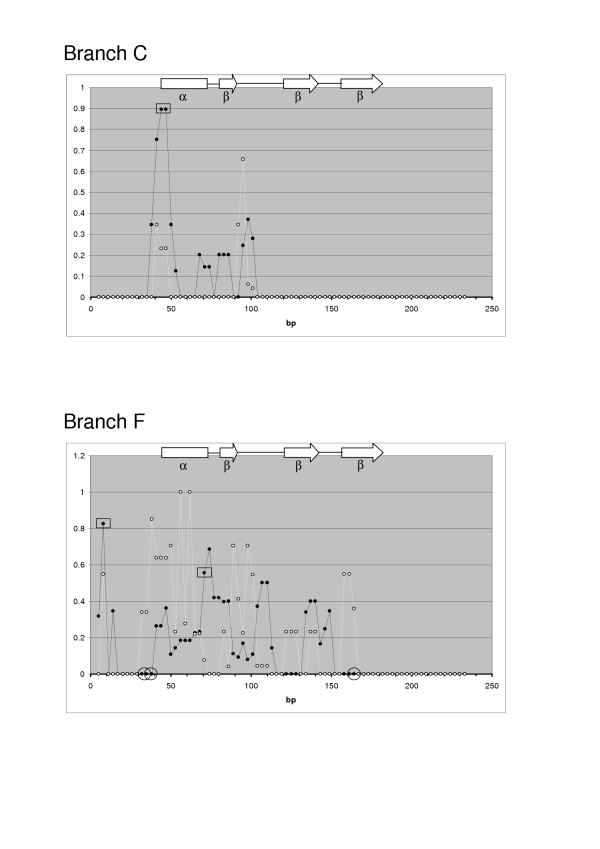
**Substitution rates and selection measured in two branches of the tree relating mammalian β-defensins. **Each graph shows Ka (black circles), Ks (white circles) and significant positive selection across the sequence (midpoints of 3 codon windows) encoding the mature peptide. The graphs display the analysis for branches C and F in Figure 1, a relatively recent and an older branch demonstrating positive selection respectively. The positions of the beta strands and alpha helix in DEFB1 are indicated.

SWAPSC found statistically significant support for positive and negative selection at many sites. Figure 3 shows the raw data graphed for two of the branches (the most recent and oldest in the tree) demonstrating significant evidence for positive selection (Figure 1). For each branch the Ka and Ks measured at successive 3 codon windows are shown. These graphs make clear that the sites of positive and negative selection identified as significant are likely to be a subset of those actually subject to these forces in reality. Figure 3 is also typical of the results obtained for relatively recent and older branches (Figures for all other branches showing significant selection are available in Additional Files.) The recent branch shows the changes between the last common ancestor of *C. aethiops *(vervet monkey) DEFB106 and *P. anubis *(olive baboon) DEFB106 and DEFB106 in *C. aethiops*. Most regions of the molecule show little or no changes, as expected over ~9.62 MY but two consecutive windows (bp 40–48 and 43–51) demonstrate a significant excess of Ka over Ks. The older branch shows changes between the last common ancestor of all primate DEFB1 and mouse Defb1 sequences and the ancestral primate DEFB1 sequence. This older branch concerns events ~40–92 MYA and shows greater variation in Ka and Ks across the sequences, though only two regions show significant evidence for positive selection (bp 4–12 and 67–75) and a further two for negative selection (bp 28–42 and 160–168). There are many sites such as this where selection is detected similarly in all or most lineages, reflecting more ancient events in mammalian evolution. All except one of the conserved cysteine residues are implicated as being under negative selection in both primate and mouse lineages. Similarly, a small region at the extreme N terminal of the mature peptides (positions 2–4 in Figure 4) was found to be under positive selection in primate and mouse lineages. However certain regions of these molecules have experienced positive and negative selection in different lineages. Arguably it is these sites, where selection has at one time favoured a change but at another required stasis, that are likely to be most potent in altering the functions of these proteins. These sites mainly cluster at a central region of the mature peptides (positions 36–40 in Figure 4), although other sites, often those neighbouring cysteine residues (positions 13–14, 24–25, 33 and 55–56), appear to have been subject to such opposing selection.

ADAPTSITE (using a likelihood approach) also nominated sites showing significant evidence of positive and negative selection and these can be compared with those nominated by SWAPSC and by PAML run separately on mouse and primate datasets (Figure 4). However there are no sites in primates and only three in mouse (positions 21, 33 and 37 in Figure 4) where all three programs show evidence for positive selection. This lack of agreement between all three programs is perhaps unsurprising considering the different restrictions of each analysis. SWAPSC can find lineage-specific events but relies on many simulated datasets to assess significance and lacks the single site resolution of the other two programs. PAML was restricted here to examining selective episodes between paralogs and does not test for significant evidence of negative selection. ADAPTSITE results are only reliable for alignments positions with more than 15 nucleotide differences [21]. In addition both ADAPTSITE and PAML do not consider gapped positions in the alignment, whereas SWAPSC will if the gap is absent from the lineage under examination. Perhaps most importantly both the PAML and ADAPTSITE analyses discussed here examine site-specific events across the whole alignment under scrutiny, effectively averaging over lineages. Figure 4 shows the level of agreement between any pair of programs and appears to indicate only modest agreement between them on the location of sites under positive selection. Most of the positively selected primate sites (5/9) and mouse sites (7/7) implicated by PAML are also supported by either SWAPSC or ADAPTSITE. Similarly most of the positively selected primate sites (2/2) and mouse sites (14/18) implicated by ADAPTSITE are supported by at least one of the other programs. Following the logic of Podlaha and Zhang [22] such sites, supported by more than one independent analysis, are the most reliably inferred. However, this assertion assumes that the three methods used are similarly informative for the present dataset. Significant heterogeneity between the results of the three methods might indicate they are not. A heterogeneity G-test [23] was used to assess uniformity between the outcomes of the three tests (SWAPSC, PAML and ADAPTSITE) for positive selection. The numbers of sites predicted to be positively selected (per 10 residue interval across the alignment in Figure 4) were counted. It was necessary to consider all positively selected sites predicted for mouse and primate data together, and to collapse the first and final intervals to create intervals containing sufficient numbers of predicted sites (i.e. greater than zero). This calculation indicates that G_H _= 11.86 with 6 degrees of freedom which is not significant. Thus although the results of the three tests for positive selection used do not agree perfectly there is no significant heterogeneity between them.

### Structural implications of evolutionary history for β-defensin peptides

**Figure 5:**
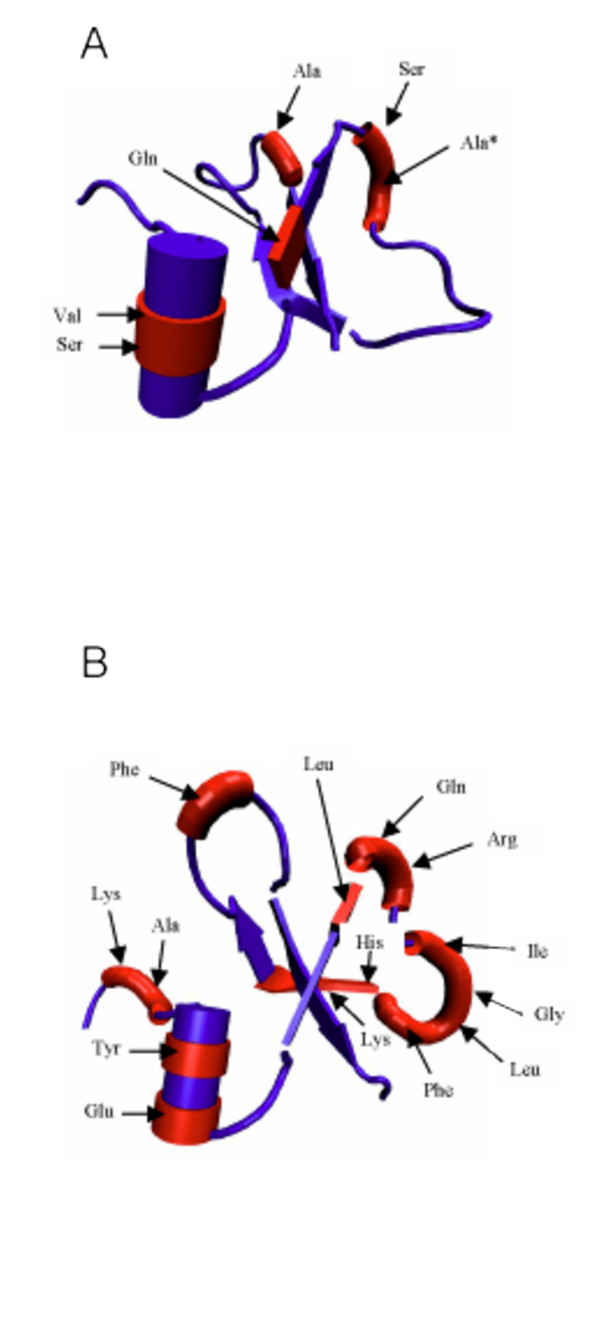
**Structural implications of primate and mouse sites under significant selection. **Each site is implicated by two or more programs from the following three: PAML, ADAPTSITE and SWAPSC. Primate sites were mapped to the structure of the human DEFB1 mature peptide (A) and mouse sites to the structure of the mouse Defb7 mature peptide (B). Sites subject to selection are depicted as inflated regions of the structures coloured red to indicate positive selection. The particular residues in DEFB1 and Defb7 corresponding to positively selected sites are also indicated with arrows. Ala (marked with an asterisk) is subject to negative and positive selection in different primate lineages.

It has been shown that primate and murine β-defensins share striking similarity at the level of secondary and tertiary structure, in spite of very low levels of sequence similarity [26]. The most reliably inferred sites of selection in Figure 4 (those implicated by more than one different method) were mapped to the known structures of the human DEFB1 and the mouse Defb7 mature peptides to examine differences in the distribution of these sites between primate and rodent lineages (Figure 5). As discussed above, there are more sites demonstrating positive selection in the murine defensins as compared to the primate defensins (Figure 4). However some clear similarities between the positions of positively selected sites are evident on the murine and primate structures (Figure 5). It seems that sites within the triple beta-strand so characteristic of these peptides are largely unaffected by positive selection. The few exceptional sites subject to positive selection found in the triple stranded β-sheets that form the structural core of the β-defensins, may represent alterations in the oligomerisation of β-defensins (Figure 5).

Both primate and rodent lineages show a large number of sites subject to positive selection within the N-terminal portion of the mature peptide. Two sites for both primate and murine, were located within a region which in DEFB1 and Defb7 forms an alpha helix. Since regions of proteins within membranes are often helical, with surfaces covered with hydrophobic resides, we speculate that the alpha helical section may be involved in anchoring the β-defensin to a bacterial cell wall. Thus the sites within the alpha helix under positive selection may be significant in the specificity of β-defensins, either with respect to their antimicrobial or chemoattractant properties. The longest loop region of these peptides (indicated to the right on Figure 5) also contains sites of positive selection. For the murine form this loop is almost exclusively subject to high selection, which suggests that this part of the structure has a key functional role in these small peptides. If, as shown for the β-defensin HNP3 [27] the second beta-strand is involved in oligerimisation, many of these sites would all be left exposed after dimer formation, suggesting an rapidly diverging exposed 'skirt' around the peptide. This is confimed by the NMR data of Schibli et al. [25] whose structures of the human β-defensin DEFB3 suggest symmetrical dimer formation, through the beta strand 2 of the β-sheet.

Some sites found to be subject to positive selection in rodents (see Figure 4) are not represented in Figure 5 as they are part of the N-terminal prepropeptide region that is removed to produce the mature peptide. This contrasts with studies of α-defensins which have found an absence of positively selected sites in the prepropeptide region [24]. In primate lineages sites within the prepropeptide region have undergone negative selection (Figure 4). These observations strongly imply that the prepropeptide region is more important to β-defensin function than has previously been appreciated.

## Discussion

The present data demonstrated evidence for positive and negative selection in different branches but at overlapping regions of the same molecules. It is clear that in such a case the use of site specific LRTs (effectively averaging across the branches of a tree) may have little power to detect the sites involved. A combined approach, using such LRTs with an independent method (SWAPSC) examining each branch individually has previously been employed in an analysis of α-defensins [24]. Here we have extended this approach to examine ancient and more recent events in primate β-defensin evolution.

It is thought that positive selection may play a major role in the divergence of paralogues from one another following a duplication event [28]. In agreement with this many of the more ancient selective episodes detected here appear to date back to the birth of these genes by duplication at an ancestral mammalian locus. However it is striking that later episodes of positive selection often focus on sites overlapping those subject to the more ancient events, particularly in the rodent lineage. This suggests that many of the same sites that originally conferred specificity of function upon these peptides were altered to provide novel functions many millions of years later. Although in the primate lineage it would appear that the original episodes of positive selection following duplication were usually followed by negative selection.

Boniotto et al. [13] found some evidence for positive selection in human DEFB4 during the divergence of primate species. They reported several residues of interest in the mature peptide where substitutions were observed in primate groups other than the great apes. These N-terminal residues are concerned with oligomerisation in the human peptide and they suggested that a particular form of oligomerisation might have evolved in apes and humans. We detected no significant positive selection in DEFB4 since the divergence of the same primate groups, although our analysis is conservative. However the only statistical evidence presented by Boniotto et al. [13] to support their hypothesis that DEFB4 is subject to positive selection was a Z-test, which is not stringent enough for short sequences such as these.

A consequence of the more rigorous statistical analyses to which we subject the sequences described here is that we may miss real episodes of positive selection. Antcheva et al. [29] synthesised variant molecules based on their observation that DEFB4 (formerly DEFB2) has been subject to positive selection during the divergence of various primate lineages. They synthesised the *M. fascicularis *DEFB4 orthologue ("mfaBD2") and a variant of the human peptide lacking Asp(4), ("-D)hBD2", which is characteristic only of the human/great ape peptides. hBD2 and mfaBD2 showed a significant difference in specificity, the former being more active towards *Escherichia coli *and the later towards *Staphylococcus aureus *and *Candida albicans*. Asp(4) in the human peptide appears to be important, as (-D)hBD2 was less structured and had a markedly lower antimicrobial activity but this site was not identified as being subject to positive selection here. A clear but unexpected result of the present analyses was that the preproregion has been subject to significant positive selection in rodents and negative selection in primates. This has not been observed previously. It is commonly assumed that the preproregion is cleaved as the mature peptide is secreted from the cell. We conclude that further investigations of cleavage and the functional consequences of sequence changes in this region are merited.

## Conclusions

We have used a combination of maximum likelihood based tests and a maximum parsimony based sliding window approach to give the most statistically rigorous and detailed view of the selective history at the major primate β-defensin locus. These data shed light on the evolution of human innate immunity but also have practical applications in the design of novel antibiotics. Sites within the active, mature peptides have been subject to repeated episodes of selection in different primate lineages, and by implication are important in functional diversity. Additional sites within the prepropeptide region, which is cleaved before secretion, were also subject to selection suggesting a previously unappreciated functional significance of this region.

## Methods

### Sequence data

In alignments and figures primate species names were abbreviated to two letters as follows: *Cercopithecus preussi *(Preuss's monkey) (cp), *Cercopithecus aethiops *(Vervet monkey) (ca), *Cercopithecus erythrogaster *(Red-bellied monkey) (ce), *Presbytis cristata *(Silvered langur) (pc), *Presbytis obscurus *(Spectacled langur) (po), *Presbytis melalophos *(Banded langur) (pm), *Macaca mulatta *(Rhesus Macaque) (mm), *Macaca fascicularis *(crab-eating macaque) (mf), *Papio anubis *(olive baboon) (pa), *Hylobates lar *(Lar gibbon) (hl), *Hylobates moloch *(Silvery gibbon) (hm), *Hylobates concolor *(crested gibbon) (hc), *Callithrix jacchus *(common marmoset) (cj), *Saguinus oedipus *(cotton-top tamarin) (so), *Pan troglodytes *(chimpanzee) (pt), *Gorilla gorilla *(gorilla) (gg), *Pongo pygmaeus *(orangutan) (pp), *Homo sapiens *(human) (hs). The Cercopithecidae are represented by cp, ca, ce, pc, po, pm, mm, mf and pa; the Hylobatidae by hl, hm and hc; the Callitrichidae by cj and so; and the Hominidae by pt, gg, pp and hs. Note that sequences from every species were not available for each primate gene (see Figure 1 and Additional Files).

All mouse sequences were recently published by Zaballos et al. [30]. The *H. sapiens *and *P. anubis *sequences were as previously published [10]. Previously published sequence data for primate DEFB1 [11], DEFB4 [13] and DEFB103 [12] were combined with the following novel data. Whole genome shotgun reads from the *M. mulatta *genome representing DEFB104 (69840222, 73807150), DEFB105 (73492526, 74381588, 72060044) and DEFB108 (71259620, 72564100, 71889652, 72776644, RHQRA66TR) were identified using BLAST [31] from the Ensembl Trace server . Genomic sequence assembly contigs from the *P. troglodytes *genome were obtained from Ensembl in the same way for DEFB105 (AADA01159356) and DEFB107 (AADA01159356). The published *Rattus norvegicus *(rat) genome assembly [32] and the full *Canis familiaris *(dog) ~7.6X coverage whole genome shotgun data (downloaded September 2004 from the Ensembl Trace Server: ) were searched using TBLASTN [31] with default settings.

PCR of novel second exon sequences from *P. anubis*, *C. aethiops*, *P. pygmaeus*,*C. jacchus *DNA was achieved using primers designed to the human exon 2 flanking sequence. PCR programmes were used with a relaxed annealing temperature that revealed a single species by gel electrophoresis. PCR products were cloned and several clones were analysed for each PCR. At least two clones were sequenced in both directions. Primers were as follows with forward primer sequence preceding the reverse primer sequence for each gene. DEFB103: 5'GTGCTGTTTTGTCATTGCAG, 5'GATTTAAAAAAAAAAATCAAGCTC; DEFB104: 5'CAGTGCCATATCCTGTTATCTAG, 5'GCTGCTAGGCCGCAGGAAGG; DEFB105: 5'GCAGCTCTTTCTTGGCAGAG, 5'GCTGGTCTGGTTTGTCAGATC; DEFB106: 5'TGGCTCCTTCCCTGTGTAG, 5'CACTTGACAAACTGAGCAAAG; DEFB107: 5'CTGCTTTCTTTACTTAGCCA, 5'GTGCTTAGTTTTTAATGTTTCTTTC; DEFB108: 5'CAATAACCCCTTCTGCATGTAG, 5'CTCAATTCTTGGTTGATGCCC. Novel sequences were deposited in GenBank under accession numbers: AY831729, AY831730, AY831731, AY831732, AY831733, AY831734, AY831735, AY831736, AY831737, AY831738, AY831739, AY831740, AY831741, AY831742, AY831743, AY831744, AY831745, AY831746.

### Phylogenetic inference and evolutionary analyses

Protein sequences were aligned using T-Coffee [33]. An alignment of coding sequences corresponding to the protein alignment was constructed using the tranalign program from the EMBOSS package [34]. A phylogenetic tree was constructed using MEGA (version 2.1 [35]) by the neighbour joining (NJ) method [36] using p-distance estimates, which is thought to be the most reliable method for constructing NJ trees of closely related sequences [37]. The tree was rooted with chicken gallinacin 1 (P46156) and the reliability of each node was assessed with 1000 bootstrap replications. All of the best supported branches (>= 50% of replications) were also observed in an equivalent NJ tree constructed with the gamma distribution model implemented to account for rate heterogeneity among sites. The shape parameter of the gamma distribution (α) was estimated using baseml from the PAML package (version 3.13 [38]). The same alignment was used as the basis for trees constructed by maximum parsimony (using MEGA version 2.1 with default settings) and maximum likelihood (using PHYLIP version 3.6 [39] with default settings). In both cases the trees produced shared substantively similar topology with the NJ trees. (All trees are available on demand.) Nodes within the primate lineage were dated according to a widely accepted phylogenetic analysis [40]. The divergence of rat and mouse was taken to be 12–24 MYA [32] and the last common ancestor of mammals was assumed to be 92 MYA [41].

Likelihood ratio tests (LRTs) were performed using codeml with the site-specific models of the PAML package and the tree constructed as above. The six site-specific models recommended by Anisimova et al. [15] were tested: M0 (one-ratio), M1 (neutral), M2 (selection), M3 (discrete), M7 (beta), and M8 (beta+ω). These LRTs indicate whether the substitutions inferred from an alignment are best explained by one of two models of ω = dN/dS, where dN and dS are the nonsynonymous and synonymous substitution rates respectively. When parameter estimates under a model allowing positive selection suggest the presence of a number of sites with ω > 1, Bayes theorem was used to calculate the posterior probability that a given site is one of those that are selected [38]. It is worth noting that PAML LRTs are reported to be conservative for short sequences, though the Bayesian prediction of sites under positive selection is largely unaffected by sequence length [15].

Concerns have been raised over the reliability of particular sites inferred to be subject to positive selection using PAML [19] and so corroborating evidence was sought from independent methods. Evidence for positively and negatively selected sites was sought using ADAPTSITE (version 1.3) according to the procedure recommended by Suzuki [21]. Specifically, equal equilibrium codon frequencies were assumed, an NJ tree based upon p-distance was used as above but was unrooted, the transition/transversion rate ratio was taken to be 1.02 for both primate and mouse datasets (estimated using MEGA) and the significance level for detecting selection was 5%. All three approaches accommodated within ADAPTSITE were employed: maximum parsimony, a distance-based Bayesian method and maximum likelihood. The maximum likelihood analysis was run with two different initial values of ω (0.00001 and 1) to ensure the results were robust to such differences, and only sites where the estimated number of nucleotide substitutions was greater than 15 were considered [21]. The selection operating in different regions of the sequences and within different branches of the phylogenetic tree under study were also estimated using SWAPSC with 1000 simulated data sets [20]. This program uses the differences between the estimated and expected numbers of synonymous and nonsynonymous substitutions to evaluate various hypotheses [42]. Firstly it seeks evidence for regions that have suffered the saturation of synonymous sites: where the number of synonymous substitutions is significantly smaller than expected. In addition it seeks mutational hotspots: regions where the number of synonymous and nonsynonymous nucleotide substitutions are greater than expected under neutrality. Remaining regions where the number of nonsynonymous nucleotide substitutions is smaller than expected (or where ω is significantly smaller than the mean ω estimated for the alignment) are identified as under negative selection. Positive selection is inferred where the estimated number of nonsynonymous nucleotide substitutions is greater than expected by chance and where ω is significantly greater than 1. Where regions have an estimated number of nonsynonymous substitutions greater than expected but ω < 1 or where ω > 1 but there is evidence for saturation of synonymous sites such regions are said to have accelerated rates of nonsynonymous substitutions. Thus SWAPSC seeks to avoid inferring positive selection where there is insufficient data to support it or where saturation may cause bias. A by-product of the SWAPSC analysis is substitution rate estimates for all branches of the tree under study, within overlapping 3 codon windows across the alignment. Each site identified by PAML or ADAPSITE as subject to selection was checked against the synonymous rate estimates made by SWAPSC to ensure that these sites were not saturated at any branch of the tree.

### Structural analysis

In order to establish the relevance of these finding to the solution structure of murine-defensins, we mapped the adaptive sites onto the nuclear magnetic resonance (NMR) structures of mouse Defb7 [26] and human DEFB1 [25]. For each structures were downloaded as PDB files from the Brookhaven protein databank, . The structures were viewed using VMD .

## Authors' contributions

CS performed the sequence analysis (in combination with PG) and statistical analyses, participated in experimental design and drafted the manuscript. AM and FK were responsible for the PCR experiments. HE and PB performed the structural analyses and helped to draft the manuscript. JD conceived of the study, and participated in its design and coordination and helped to draft the manuscript. All authors read and approved the final manuscript.

## Supplementary Material

Additional File 1 The full protein multiple sequence alignment for all primate and mouse sequences analysed, it is presented in FASTA format followed by interleaved (CLUSTALW) format. Click here for file

Additional File 2 The full nucleotide multiple sequence alignment (derived form the corresponding protein alignment in Additional file 1) for all primate and mouse sequences analysed, it is presented in FASTA format followed by interleaved (CLUSTALW) format. Click here for file

Additional File 3 Primate substitution rates and selection measured in various branches of the tree relating mammalian β-defensins (see Figure 1). Each graph shows Ka (black circles), Ks (white circles) and significant selection (rectangles for positive selection and circles for negative selection) within sliding SWAPSC windows of 3 codons across the sequence encoding the mature peptide. Graphs A, B, C, D, E and F correspond to branches A, B, C, D, E and F respectively in Figure 1. Click here for file

Additional File 4 *M. musculus *substitution rates and selection measured in various branches of the tree relating mammalian β-defensins (see Figure 1). Each graph shows Ka (black circles), Ks (white circles) and significant selection (rectangles for positive selection and circles for negative selection) within sliding SWAPSC windows of 3 codons across the sequence encoding the mature peptide. Graphs G, H, I, J, K, L, M, N, O, P and Q correspond to branches G, H, I, J, K, L, M, N, O, P and Q respectively in Figure 1. Click here for file

Additional File 5 Phylogenetic tree relating primate and mouse β-defensin proteins constructed using neighbour-joining. Identical to Figure 1 but with the addition of bootstrapping support (above branches) and branch lengths (below branches). Primate species names are abbreviated as detailed in Materials and Methods, mouse genes are in lower case. Click here for file
