# Supplementary figures and images for: The complexity of selection at the major primate β-defensin locus
Source: BMC Evol Biol. 2005 May 18;5:32. doi: 10.1186/1471-2148-5-32 (PMC1156880; doi:10.1186/1471-2148-5-32)

A

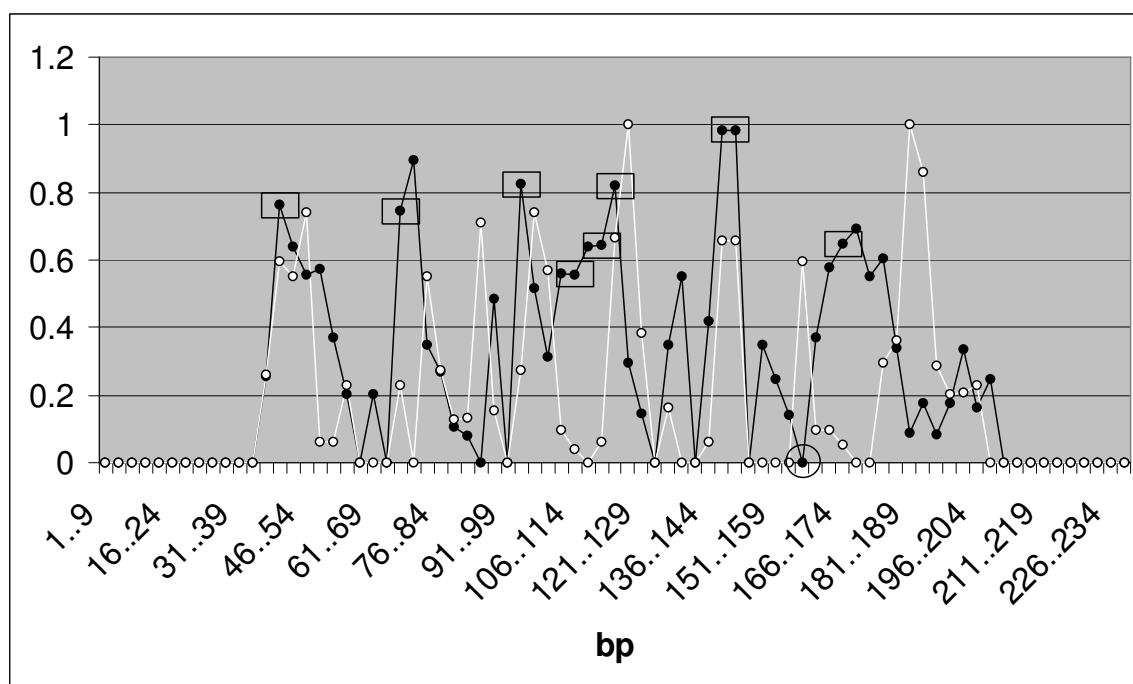

B

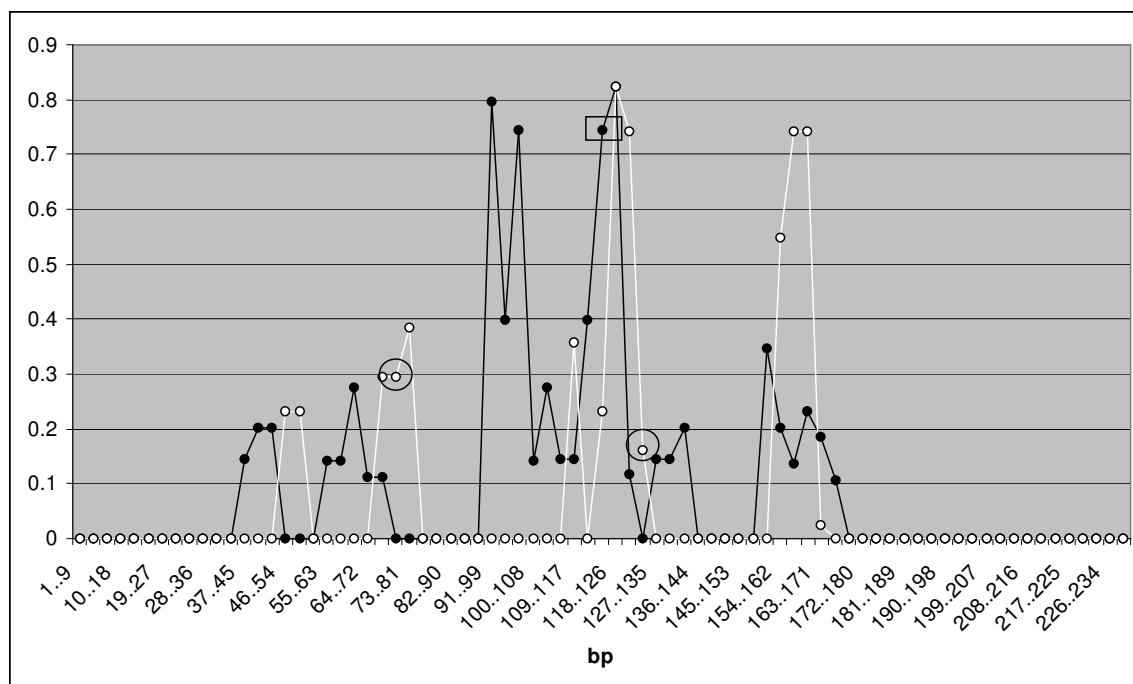

C

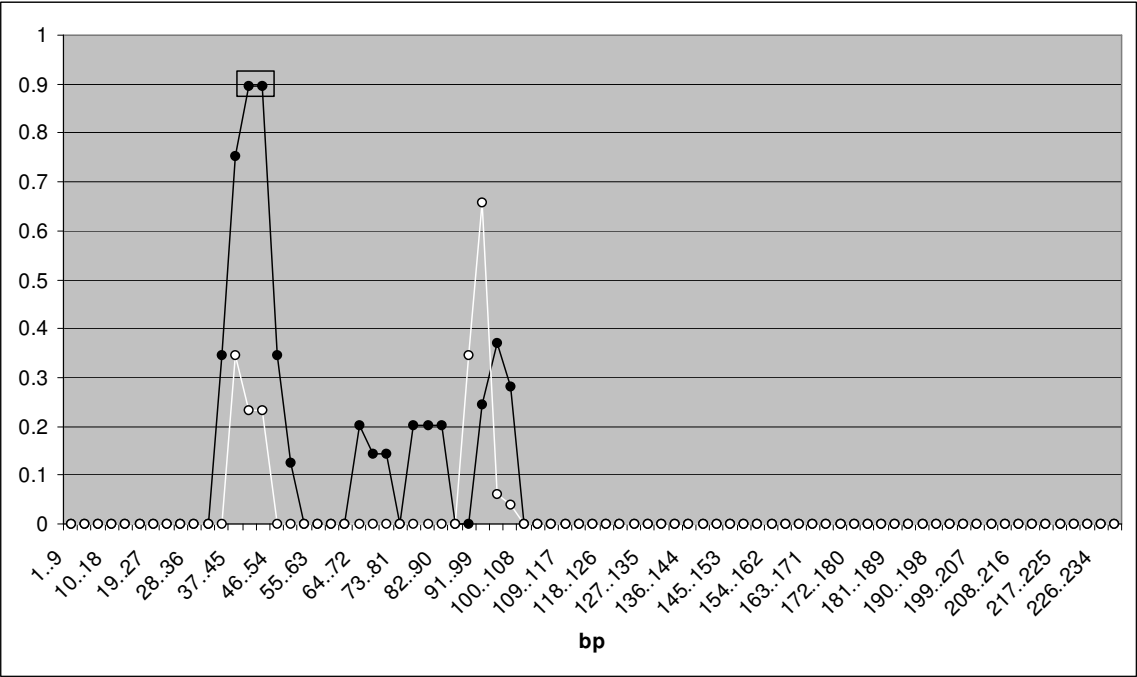

D

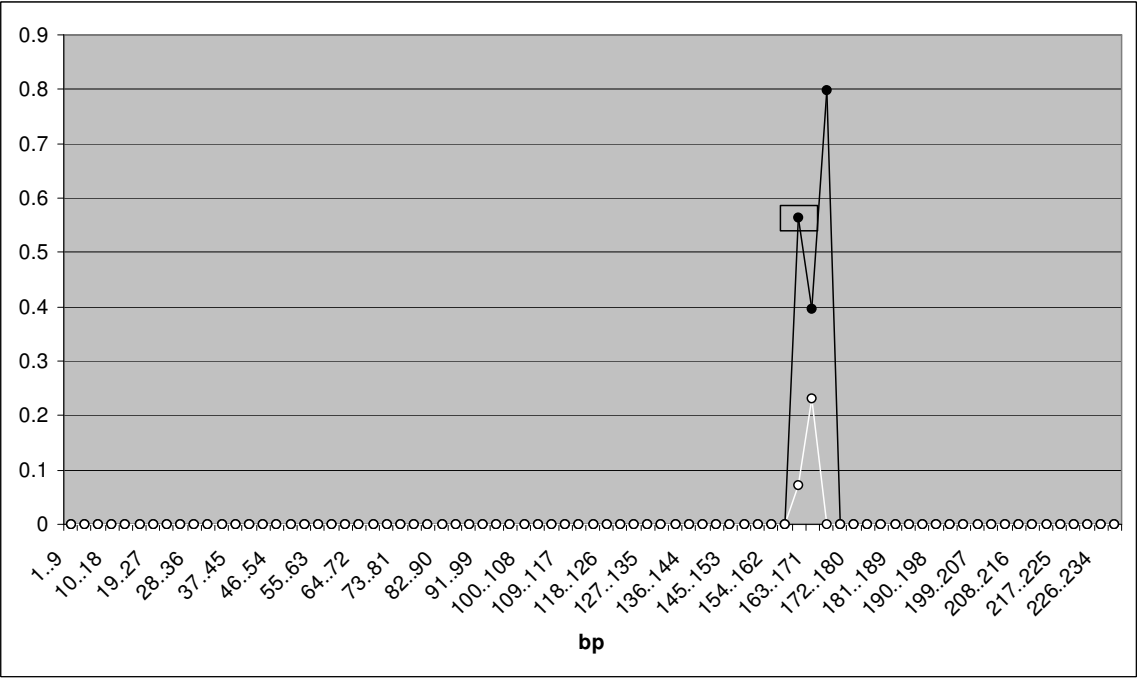

E

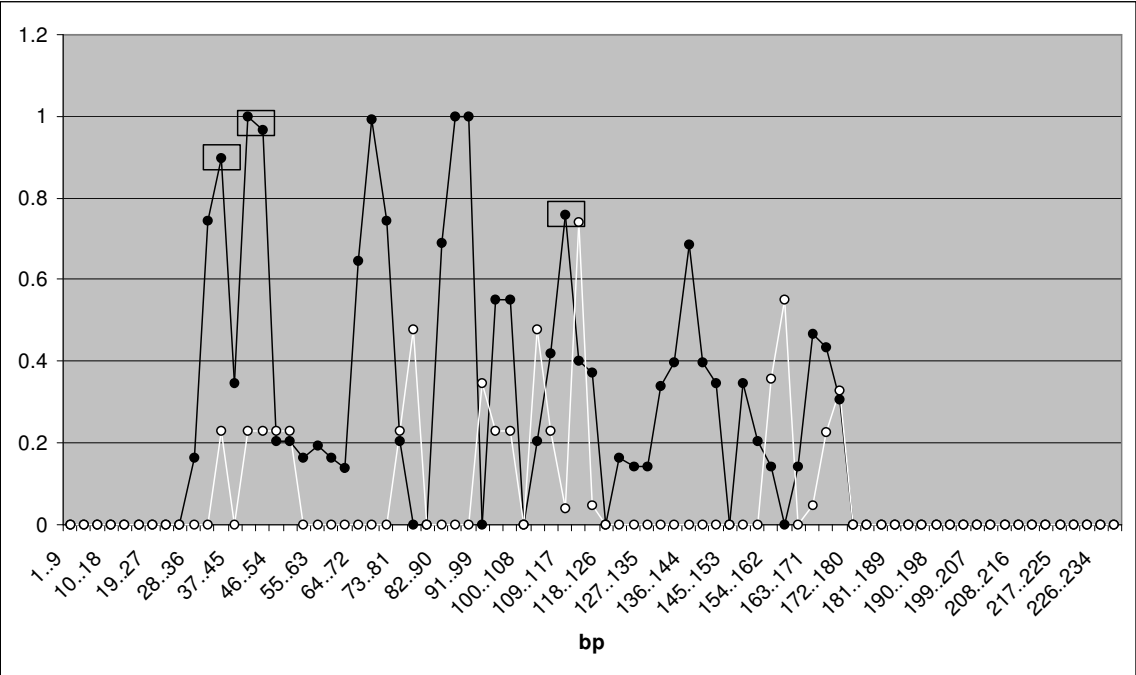

F

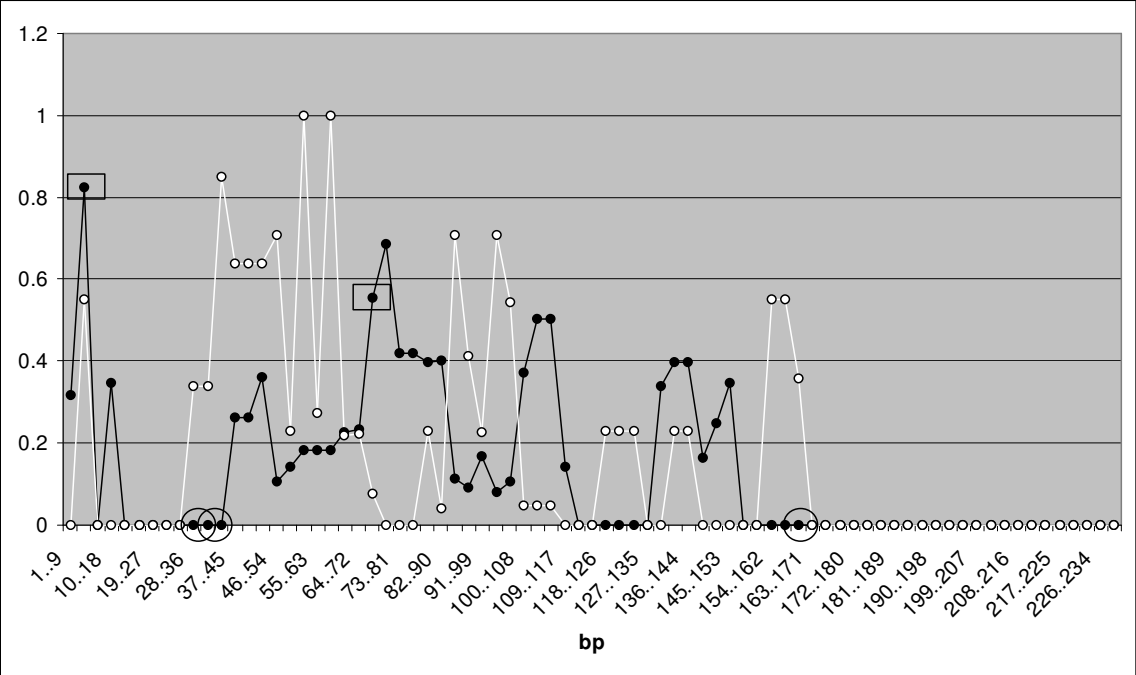

Supplement: Additional File 3 — Primate substitution rates and selection measured in various branches of the tree relating mammalian β-defensins (see Figure 1). Each graph shows Ka (black circles), Ks (white circles) and significant selection (rectangles for positive selection and circles for negative selection) within sliding SWAPSC windows of 3 codons across the sequence encoding the mature peptide. Graphs A, B, C, D, E and F correspond to branches A, B, C, D, E and F respectively in Figure 1. [file 1471-2148-5-32-S3.pdf]

G

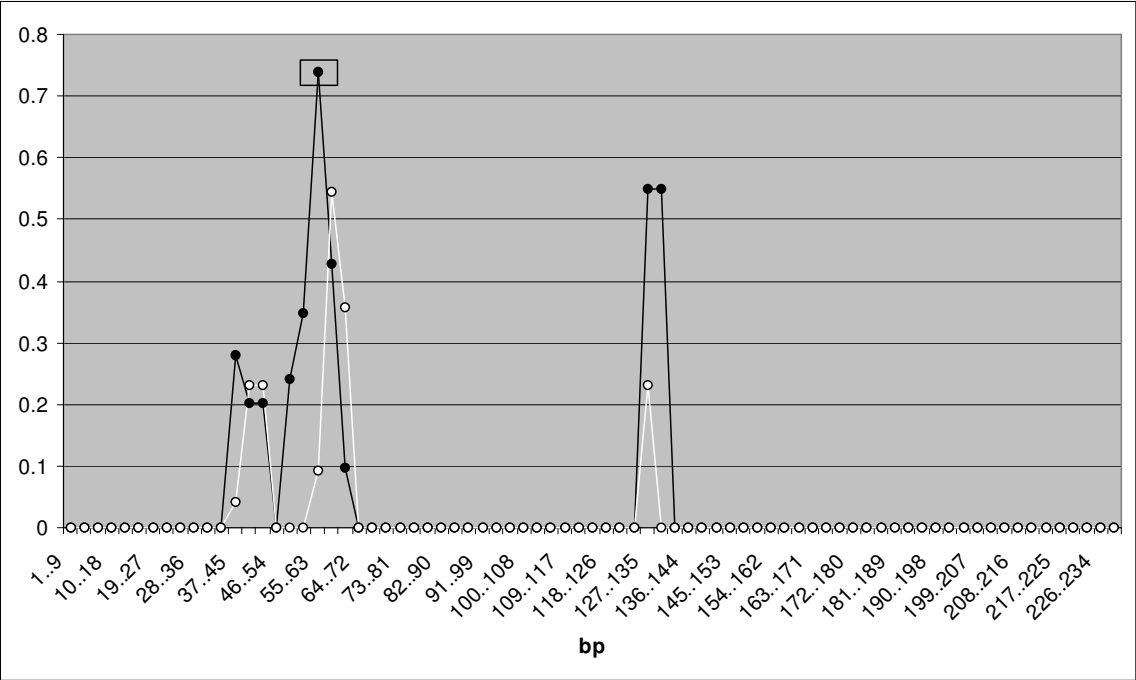

H

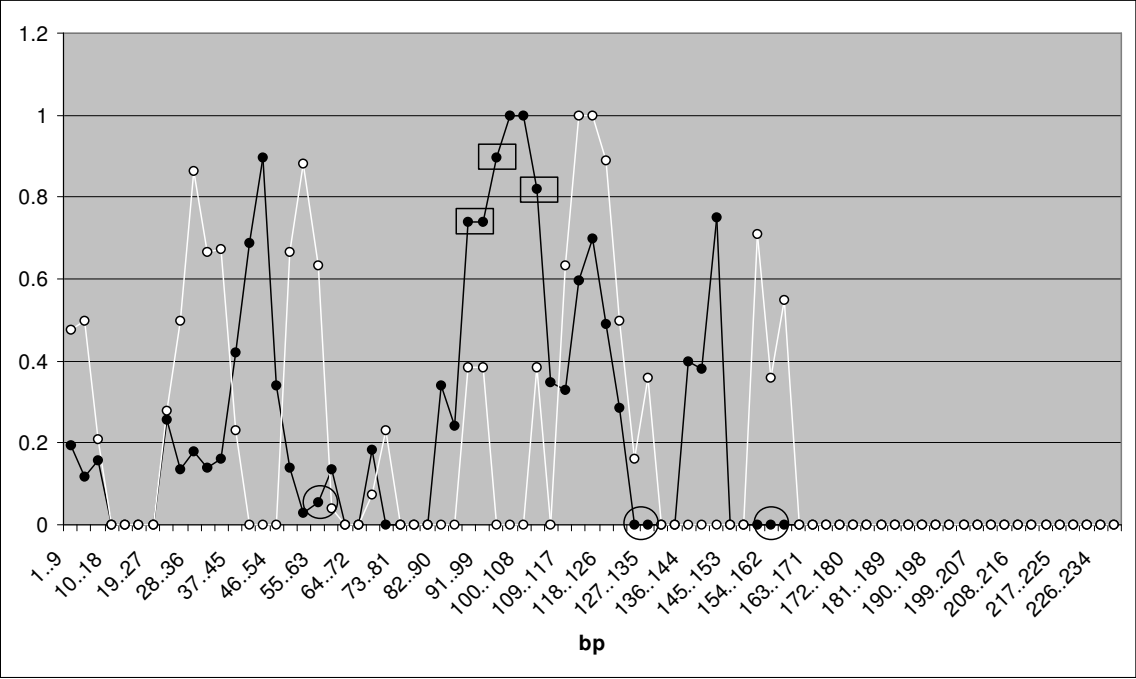

I

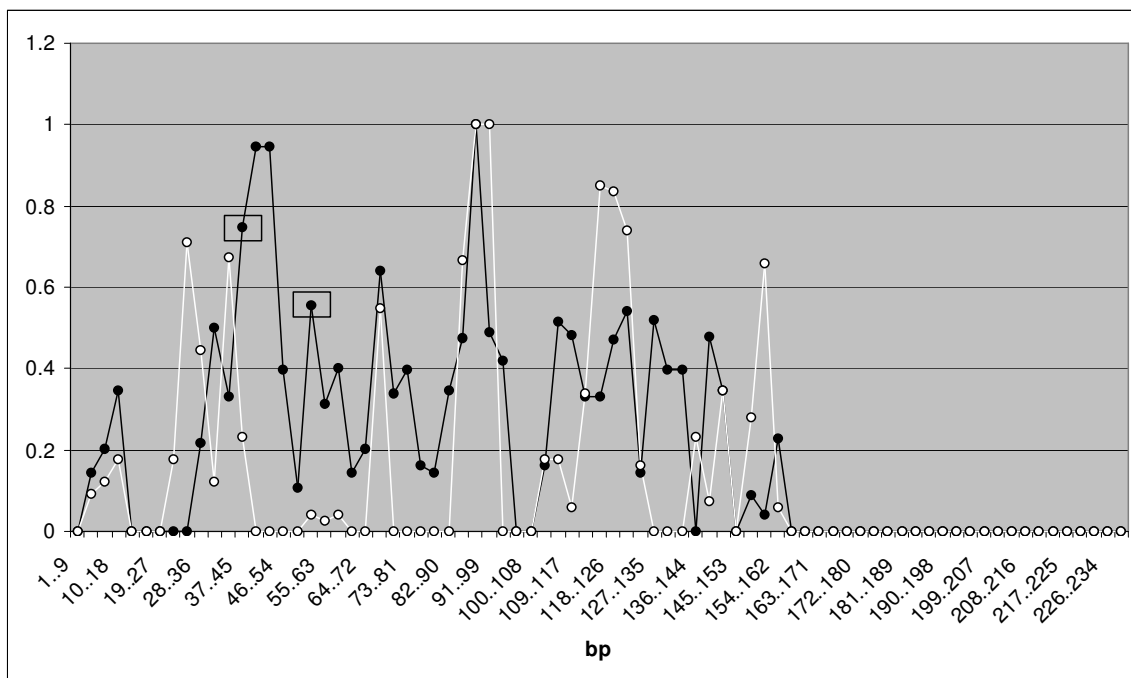

J

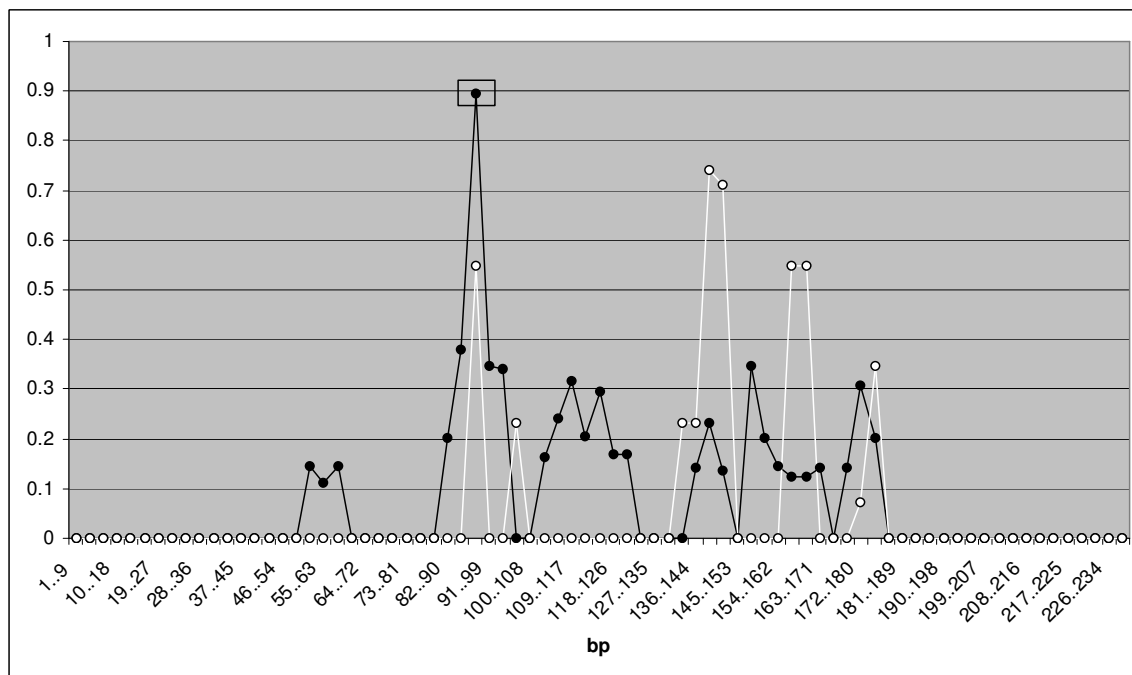

K

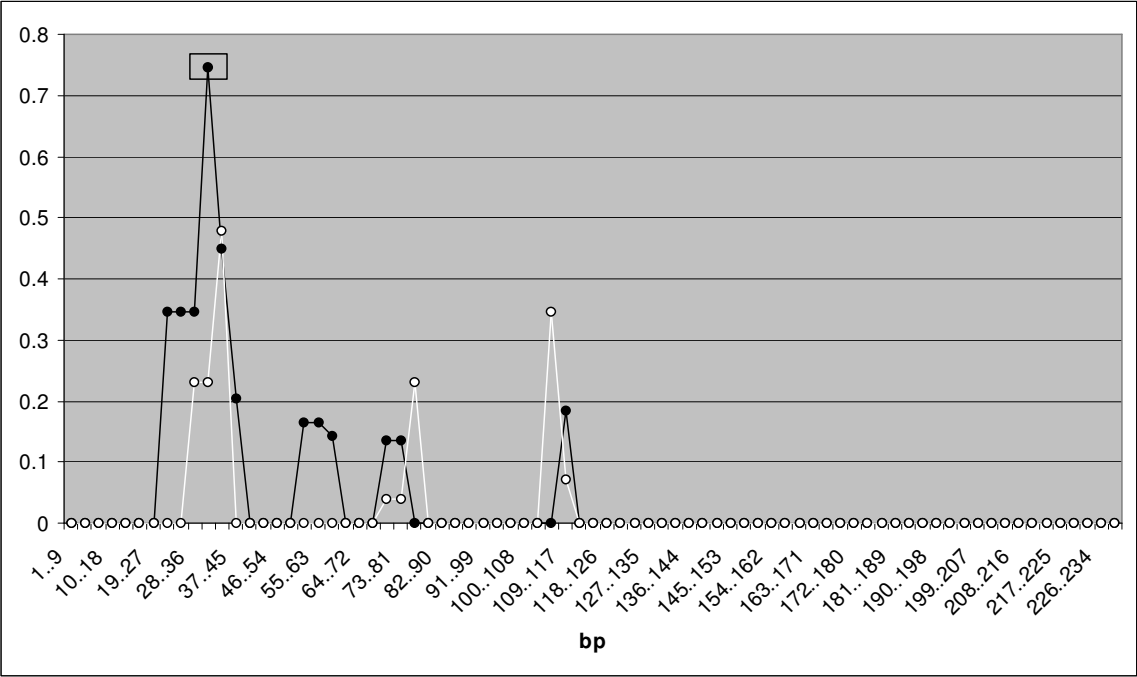

L

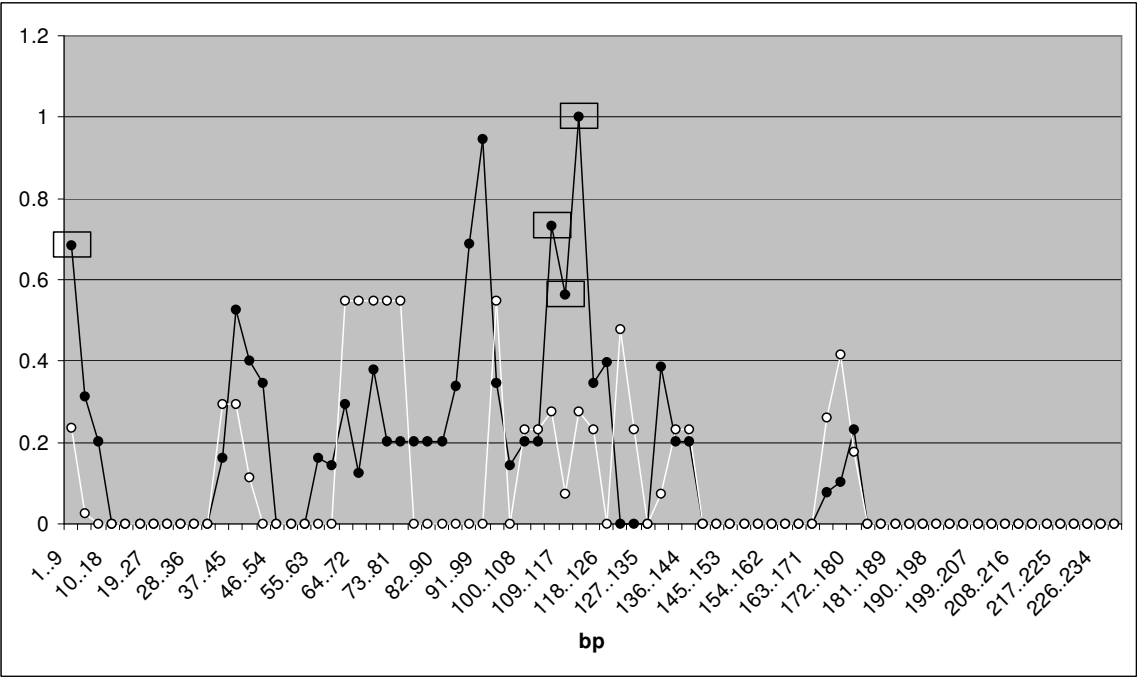

M

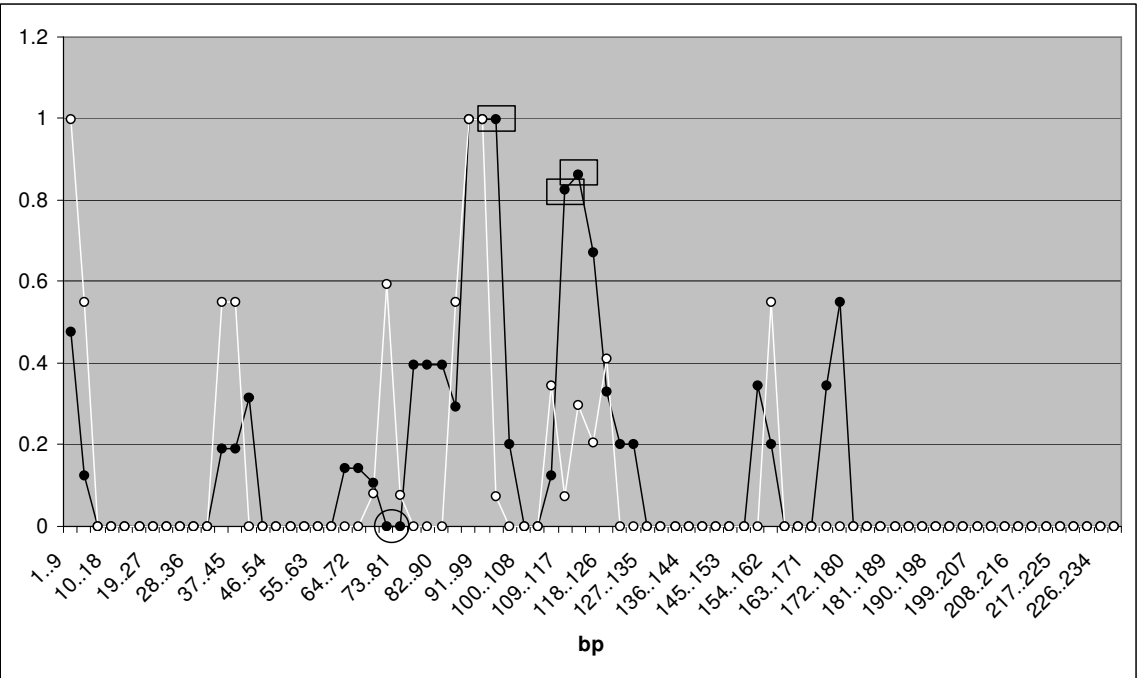

N

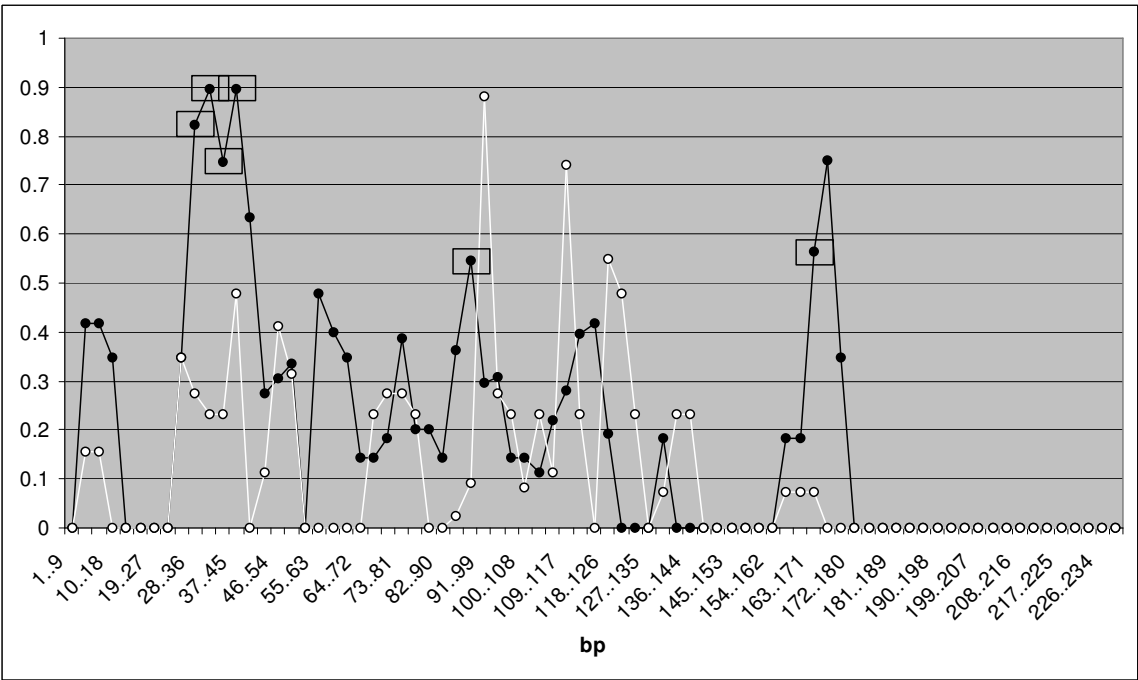

O

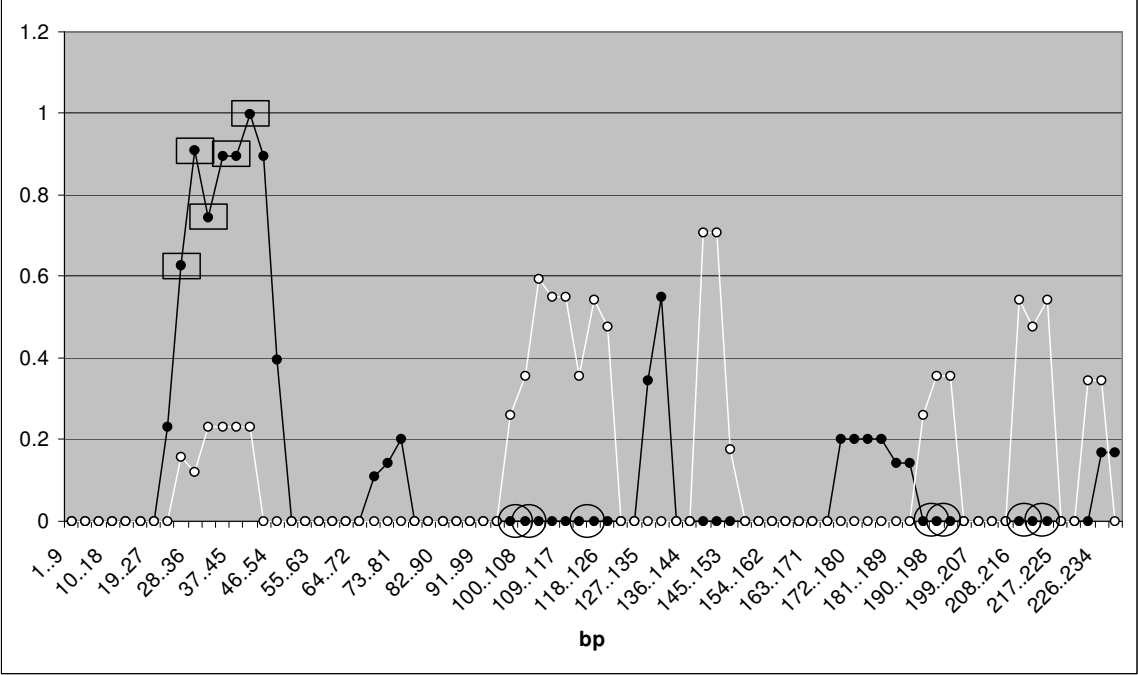

P

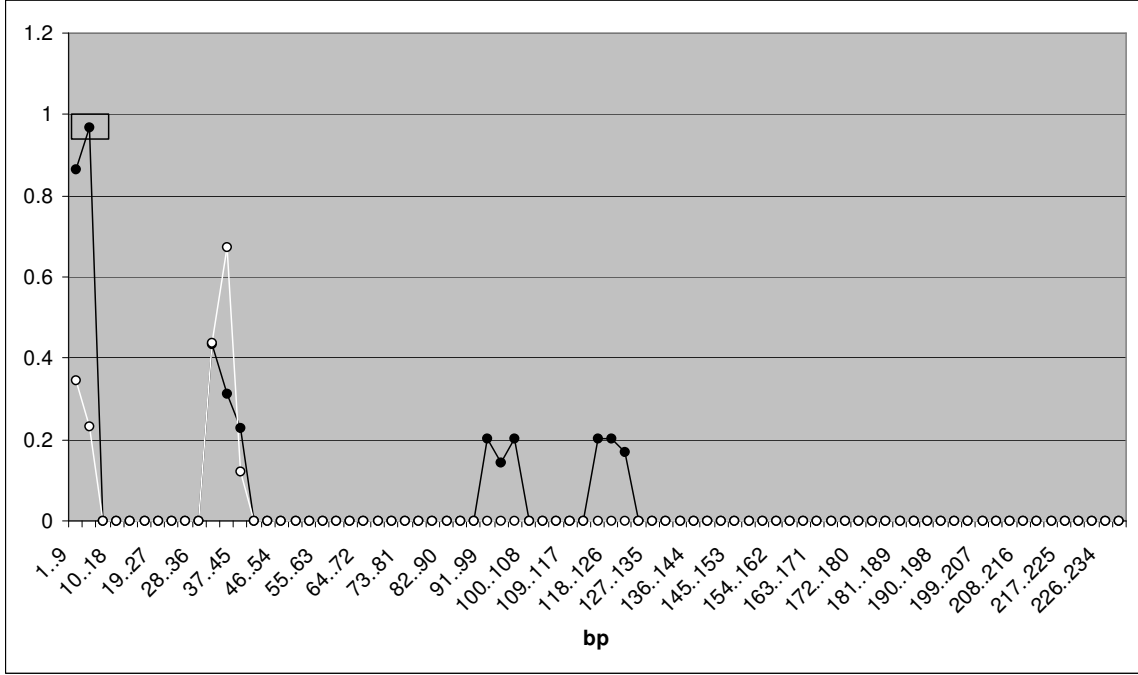

Q

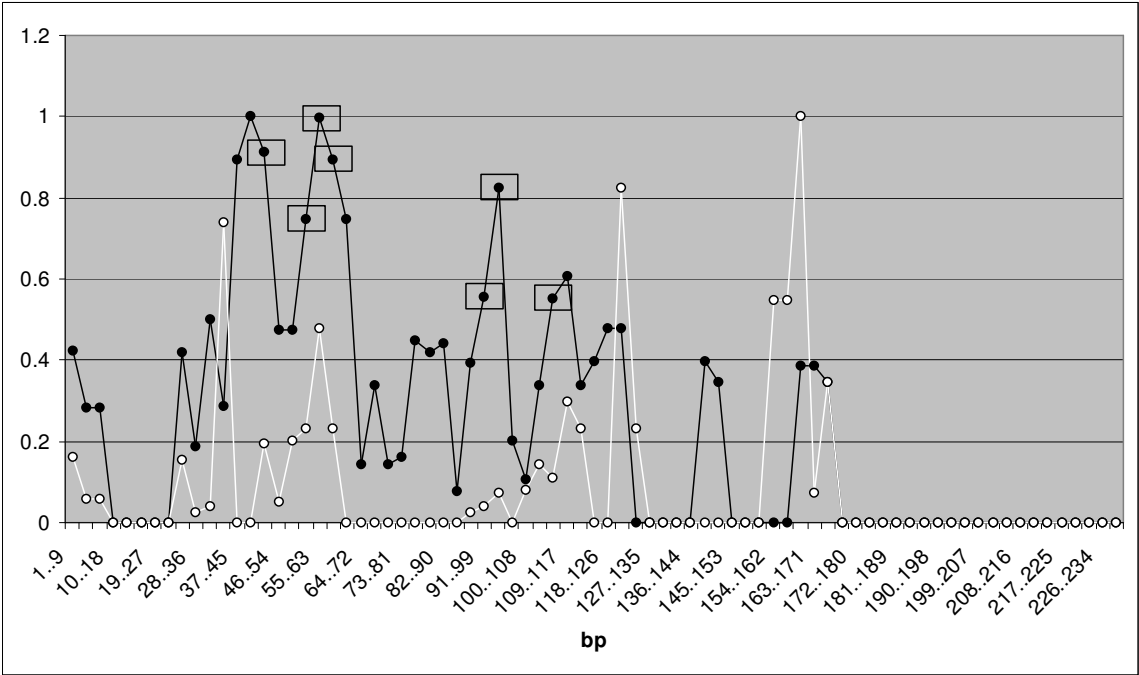

Supplement: Additional File 4 — M. musculus substitution rates and selection measured in various branches of the tree relating mammalian β-defensins (see Figure 1). Each graph shows Ka (black circles), Ks (white circles) and significant selection (rectangles for positive selection and circles for negative selection) within sliding SWAPSC windows of 3 codons across the sequence encoding the mature peptide. Graphs G, H, I, J, K, L, M, N, O, P and Q correspond to branches G, H, I, J, K, L, M, N, O, P and Q respectively in Figure 1. [file 1471-2148-5-32-S4.pdf]

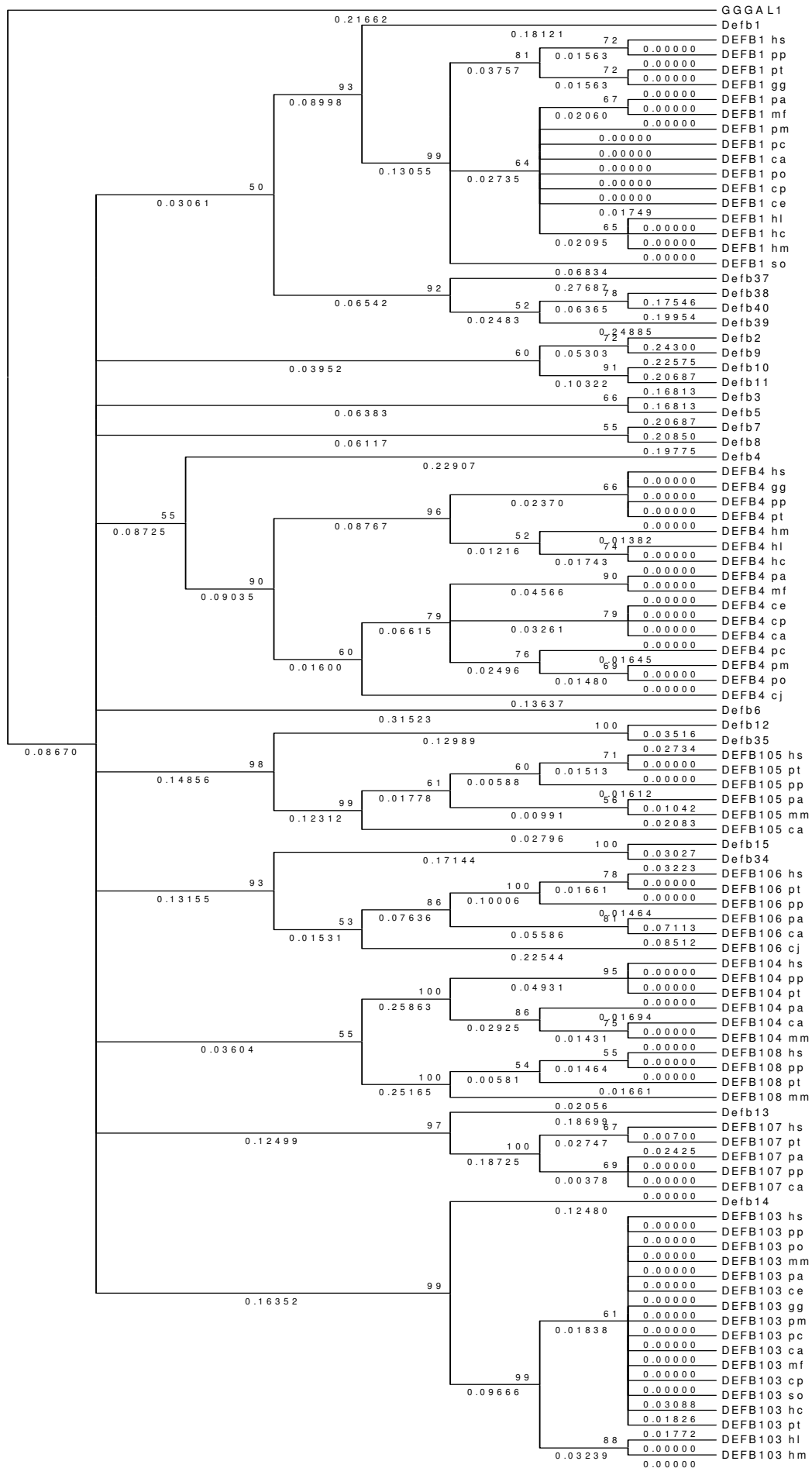

Supplement: Additional File 5 — Phylogenetic tree relating primate and mouse β-defensin proteins constructed using neighbour-joining. Identical to Figure 1 but with the addition of bootstrapping support (above branches) and branch lengths (below branches). Primate species names are abbreviated as detailed in Materials and Methods, mouse genes are in lower case. [file 1471-2148-5-32-S5.pdf]
